# Supplementary material for: The anthropometric and physical qualities of women’s rugby league Super League and international players; identifying differences in playing position and level
Source: PLoS One. 2022 Jan 31;17(1):e0249803. doi: 10.1371/journal.pone.0249803 (PMC8803183; doi:10.1371/journal.pone.0249803)
Supplement: Attachment — Submitted filename: RLW Reviewer Comments.docx [file pone.0249803.s002.docx]

Overall, I think the goal of this study is good, and adds to the limited body of research in women’s team sports. I am unsure about the aim of this study addressing the “evidence gap” around the quantification of anthropometric and physical qualities of female rugby league players, though. I agree that there is limited research in women’s rugby league, but as pointed out in your introduction this isn’t the first study to look at anthropometric or physical qualities. Your paper does a good job at adding to the previous literature by increasing the sample size of the cohort and including comparisons between international and women’s super league (WSL), but provides me with little new information than what existing evidence already does to address this “evidence gap”.

The inclusion and explanation of all procedures performed in the battery testing are exceptional, and provide quality information for others trying to replicate these tests. However, you mention in your procedures for aerobic capacity that a reduced shuttle distance was used to account for physiological differences between men and women; has this reduction in shuttle distance been quantified previously, or did you use do any internal validation for this? Do you also have a reference highlighting previous men and women’s physiological differences?

In regard to general writing structure, the paper is well structured and written.

**Specific comments are provided below.**

L101	Be consistent with how you describe playing standard. Previously and further into the paper you use the term international, yet here you say national side.

L178	Do you have a reference for physiological differences between males and females.

L180 Statistical analysis: Where international players included in both competition levels (i.e. international and WSL), are the international group just a subsample of your overall dataset? if so, how did you account for this in your statistical analysis? Do you think this would affect your results?

Did you also consider having the individual athlete as a random effect to account for potential individual differences, not just positional?

Table 2. The first two rows in your table are forwards and backs, then followed by the competition level and positions within those competition levels. It is unclear what those first two positions are highlighting? Are they the combination of WSL and international players, or? And if so how is the average height of those forwards (row 1 in table) significantly lower than other forwards listed in the table?

Table 3. In the heading you include (int) I assume as an abbreviation for international but then don’t use it here in this table or anywhere else in previous tables or figures nor within text. Is it necessary?

Table 3. I was surprised by the inclusion of cross positional analysis across different competition levels (i.e. International backs v WSL forwards). From your statistical analysis section, you highlight position, playing standard and then position*playing standard as fixed effects, but it might be worth clearly stepping through and outlining all of the different analyses that you have completed to ensure you cover off the statistical approach for everything.
